# Supplementary material for: Experimental validation of the free-energy principle with in vitro neural networks
Source: Nat Commun. 2023 Aug 7;14:4547. doi: 10.1038/s41467-023-40141-z (PMC10406890; doi:10.1038/s41467-023-40141-z)
Supplement: Supplementary file 1 — Supplementary Information [file 41467_2023_40141_MOESM1_ESM.pdf]

Supplementary Information for

Experimental validation of the free-energy principle with *in vitro* neural networks

Takuya Isomura[1*], Kiyoshi Kotani[2], Yasuhiko Jimbo[3], Karl J. Friston[4,5]

[1] Brain Intelligence Theory Unit, RIKEN Center for Brain Science, 2-1 Hirosawa, Wako, Saitama 351-0198, Japan

[2] Research Center for Advanced Science and Technology, The University of Tokyo, 4-6-1 Komaba, Meguro-ku, Tokyo 153-8904, Japan

[3] Department of Precision Engineering, School of Engineering, The University of Tokyo, 7-3-1 Hongo, Bunkyo-ku, Tokyo 113-8656, Japan

[4] Wellcome Centre for Human Neuroimaging, Queen Square Institute of Neurology, University College London, London, WC1N 3AR, UK

[5] VERSES AI Research Lab, Los Angeles, CA 90016, USA

* Corresponding author email: takuya.isomura@riken.jp

This PDF file includes:

Supplementary Figure 1

Supplementary Figure 2

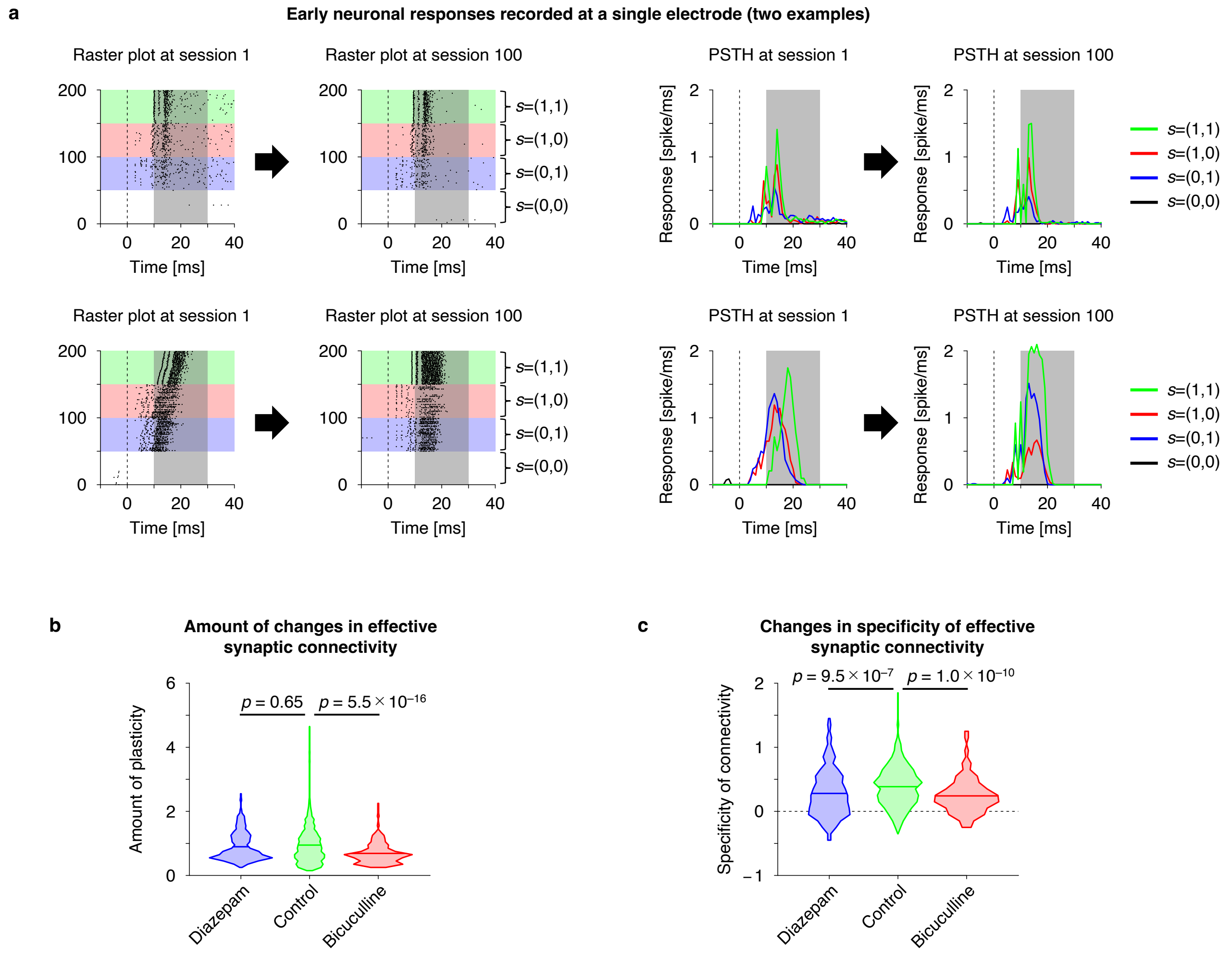

Supplementary Fig. 1. Supplementary data. a Early evoked responses of *in vitro* neurons recorded at a single electrode. Two examples are shown. **b** Amount of changes in effective synaptic connectivity, indicating the occurrence of synaptic plasticity during the training period. The amount of plasticity is characterised by the connectivity change over the training period, defined as $\sum_{k=2}^{100}\left|W_{ij}^{(k)} - W_{ij}^{(k-1)}\right|$, where $W^{(k)} = \left\{W_{ij}^{(k)}\right\}$ is a 2×32 matrix of the effective synaptic connectivity at session k and $|\cdot|$ denotes the absolute value. Distributions obtained from *n* = 448, 1920, and 384 connections (from 7, 30, and 6 independent experiments) are shown for diazepam, control, and bicuculline conditions, respectively. Horizontal bars report the mean. **c** Changes in the specificity of effective synaptic connectivity. Here, the specificity is defined as $W_{1j} - W_{2j}$ for $j = 1, \ldots, 16$ and $W_{2j} - W_{1j}$ for $j = 17, \ldots, 32$. This computes how much the contribution of a sensory electrode differs between sources 1- and 2-preferring ensembles. Distributions obtained from *n* = 224, 960, and 192 connection pairs are shown. In (b)(c), horizontal bars report the mean, and the two-sided Mann–Whitney *U* test was used for unpaired comparisons. Please refer to Methods for the definition of W.

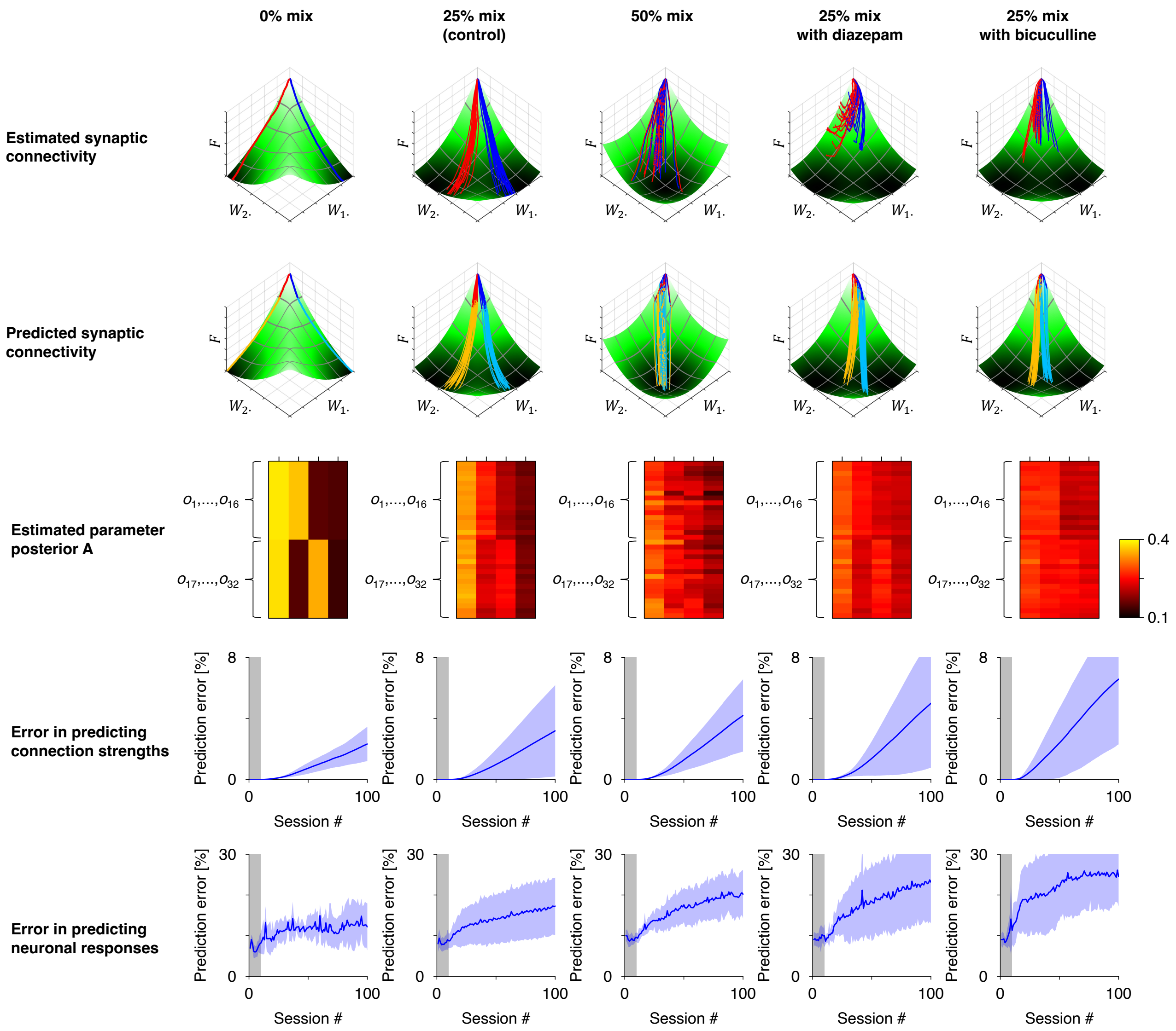

Supplementary Fig. 2. Reverse engineering of generative models and predictions of self-organisations under various conditions. Mixing matrix A in the external milieu and state prior employed by neuronal networks were varied. Top line: Estimation of synaptic trajectories. Second line: Prediction of subsequent self-organisation. Third line: Reconstruction of posterior expectation about mixing matrix A. Averages among each group are shown. Fourth line: Error in predicting synaptic strengths. Bottom line: Error in predicting neuronal responses. Prediction errors in bicuculline- and diazepam-treated groups were slightly larger than those in the control group. This may be partly because, compared with controls, the former groups involved a larger error in estimating the initial conditions. Lines and shaded areas represent mean values +/– standard deviations.
