# Supplementary material for: Experimental validation of the free-energy principle with in vitro neural networks
Source: Nat Commun. 2023 Aug 7;14:4547. doi: 10.1038/s41467-023-40141-z (PMC10406890; doi:10.1038/s41467-023-40141-z)
Supplement: Supplementary file 3 — Description of Additional Supplementary Files [file 41467_2023_40141_MOESM3_ESM.pdf]

File name: Supplementary Movie 1

Description: Trajectories of empirically estimated synaptic connectivity (left) and theoretically predicted synaptic connectivity (right) depicted on the landscape of variational free energy. Here, neuronal networks trained under three mixing conditions (25%, 0%, and 50%) are shown. Please refer to the main text and legend of **Fig. 3b,k** for details.
