# Supplementary material for: Experimental validation of the free-energy principle with in vitro neural networks
Source: Nat Commun. 2023 Aug 7;14:4547. doi: 10.1038/s41467-023-40141-z (PMC10406890; doi:10.1038/s41467-023-40141-z)
Supplement: Supplementary file 5 — Reporting Summary [file 41467_2023_40141_MOESM5_ESM.pdf]

nature portfolio

Corresponding author(s): Takuya Isomura

Last updated by author(s): Jun 5, 2023

Reporting Summary

Nature Portfolio wishes to improve the reproducibility of the work that we publish. This form provides structure for consistency and transparency in reporting. For further information on Nature Portfolio policies, see our Editorial Policies and the Editorial Policy Checklist.

Statistics

For all statistical analyses, confirm that the following items are present in the figure legend, table legend, main text, or Methods section.

n/a	Confirmed	
☐	☒	The exact sample size (*n*) for each experimental group/condition, given as a discrete number and unit of measurement
☐	☒	A statement on whether measurements were taken from distinct samples or whether the same sample was measured repeatedly
☐	☒	The statistical test(s) used AND whether they are one- or two-sided<br>*Only common tests should be described solely by name; describe more complex techniques in the Methods section.*
☐	☒	A description of all covariates tested
☐	☒	A description of any assumptions or corrections, such as tests of normality and adjustment for multiple comparisons
☐	☒	A full description of the statistical parameters including central tendency (e.g. means) or other basic estimates (e.g. regression coefficient) AND variation (e.g. standard deviation) or associated estimates of uncertainty (e.g. confidence intervals)
☐	☒	For null hypothesis testing, the test statistic (e.g. *F*, *t*, *r*) with confidence intervals, effect sizes, degrees of freedom and *P* value noted<br>*Give P values as exact values whenever suitable.*
☐	☒	For Bayesian analysis, information on the choice of priors and Markov chain Monte Carlo settings
☒	☐	For hierarchical and complex designs, identification of the appropriate level for tests and full reporting of outcomes
☐	☒	Estimates of effect sizes (e.g. Cohen's *d*, Pearson's *r*), indicating how they were calculated

Our web collection on statistics for biologists contains articles on many of the points above.

Software and code

Policy information about availability of computer code

Data collection	The data acquisition was conducted using LabVIEW version 2011.
Data analysis	The simulations and analyses were conducted using MATLAB version R2020a. The scripts are available at GitHub https://github.com/takuyaisomura/reverse_engineering. The scripts are covered under the GNU General Public License v3.0.

For manuscripts utilizing custom algorithms or software that are central to the research but not yet described in published literature, software must be made available to editors and reviewers. We strongly encourage code deposition in a community repository (e.g. GitHub). See the Nature Portfolio guidelines for submitting code & software for further information.

Data

Policy information about availability of data

All manuscripts must include a data availability statement. This statement should provide the following information, where applicable:

- Accession codes, unique identifiers, or web links for publicly available datasets
- A description of any restrictions on data availability
- For clinical datasets or third party data, please ensure that the statement adheres to our policy

Source data are provided with this paper. The neuronal response data are available at GitHub https://github.com/takuyaisomura/reverse_engineering.

Human research participants

Policy information about studies involving human research participants and Sex and Gender in Research.

Reporting on sex and gender	N/A
Population characteristics	N/A
Recruitment	N/A
Ethics oversight	N/A

Note that full information on the approval of the study protocol must also be provided in the manuscript.

Field-specific reporting

Please select the one below that is the best fit for your research. If you are not sure, read the appropriate sections before making your selection.

- [x] Life sciences
- [ ] Behavioural & social sciences
- [ ] Ecological, evolutionary & environmental sciences

For a reference copy of the document with all sections, see nature.com/documents/nr-reporting-summary-flat.pdf

Life sciences study design

All studies must disclose on these points even when the disclosure is negative.

Sample size	No methods were used to predetermine sample sizes. Sample sizes were based on experimental knowledge collected in previous works done in the lab (Jimbo et al., 1999; Isomura et al., 2015).
Data exclusions	Cell cultures with low activity levels were not used for experiments. In newly conducted experiments, 7 samples in the control condition, 7 treated with bicuculline, and 7 with diazepam were recorded. Among them, 1 sample treated with bicuculline was excluded from analyses because it exhibited abnormal activities due to disturbances in the recording environment. All published data in previous work (Isomura et al., 2015) under the control (23 samples), APV-treated (9), 0% mix (4), and 50% mix (4) conditions were involved in analyses.
Replication	In this work, 21 independent cell cultures were used for the control condition to conduct 30 independent experiments, 6 were treated with bicuculline, 7 with diazepam, 9 with APV, 4 were trained under the 0% mix condition, and 4 under the 50% mix condition. The analysis results in previous work (Isomura et al., 2015) were replicated with data obtained by newly conducted experiments.
Randomization	Cell cultures and stimulation patterns were randomly determined for each independent experiment.
Blinding	The investigators were blinded to group allocation during data collection and analysis.

Reporting for specific materials, systems and methods

We require information from authors about some types of materials, experimental systems and methods used in many studies. Here, indicate whether each material, system or method listed is relevant to your study. If you are not sure if a list item applies to your research, read the appropriate section before selecting a response.

Materials & experimental systems

n/a	Involved in the study	
x		Antibodies
x		Eukaryotic cell lines
x		Palaeontology and archaeology
	x	Animals and other organisms
x		Clinical data
x		Dual use research of concern

Methods

n/a	Involved in the study	
x		ChIP-seq
x		Flow cytometry
x		MRI-based neuroimaging

Animals and other research organisms

Policy information about studies involving animals; ARRIVE guidelines recommended for reporting animal research, and Sex and Gender in Research

Laboratory animals	19-day-old embryos (E19) obtained from Pregnant Wistar rats (Charles River Laboratories, Yokohama, Japan) were used for preparing dissociated cultures.
Wild animals	This work did not involve wild animals.
Reporting on sex	The cerebral cortices of both male and female E19 rat embryos were used for preparing dissociated cultures.
Field-collected samples	This work did not involve samples collected from the field.
Ethics oversight	All animal experiments were performed with the approval of the animal experiment ethics committee at the University of Tokyo (approval number C-12-02, KA-14-2) and according to the University of Tokyo guidelines for the care and use of laboratory animals.

Note that full information on the approval of the study protocol must also be provided in the manuscript.
